# On-Line Thermally Induced Evolved Gas Analysis: An Update—Part 2: EGA-FTIR

**DOI:** 10.3390/molecules27248926

**Published:** 2022-12-15

**Authors:** Giuseppina Gullifa, Laura Barone, Elena Papa, Stefano Materazzi, Roberta Risoluti

**Affiliations:** Department of Chemistry, “Sapienza” Università di Roma, 00185 Rome, Italy

**Keywords:** EGA, evolved gas analysis, FTIR, infrared spectroscopy, EGA-FTIR, TG-FTIR

## Abstract

The on-line thermally induced evolved gas analysis (OLTI-EGA) is widely applied in many different fields. Aimed to update the applications, our group has systematically collected and published examples of EGA characterizations. Following the recently published review on EGA-MS applications, this second part reviews the latest applications of Evolved Gas Analysis performed by on-line coupling heating devices to infrared spectrometers (EGA-FTIR). The selected 2019, 2020, 2021 and early 2022 references are collected and briefly described in this review; these are useful to help researchers to easily find applications that are sometimes difficult to locate.

## 1. Introduction

The on-line thermally induced evolved gas analysis (OLTI-EGA) can be performed by several coupled techniques to describe and often to prove a supposed decomposition mechanism by the characterization of the gaseous products released in a thermally induced reaction.

Several advantages can be achieved when the releasing process or the decomposition mechanism can be proved. Both mass spectrometry (MS) and infrared spectroscopy (IR) techniques are usually on-line, coupled by researchers, and periodic reviews have been published by our group to report the selected advances in EGA techniques and their specific applications in 1997 and 1998 [1,2], in 2001 [3,4], in 2006 [5,6], in 2010 and 2011 [7,8], in 2013 [9], in 2017 [10] and in 2019 [11].

**Figure 1:**
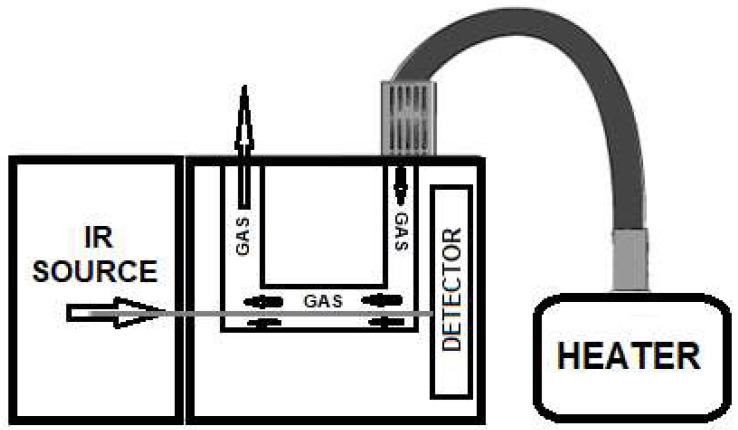
Schematic example of an EGA-FTIR system.

Gas evolution can be easily obtained by a generic “heater” that is coupled by a heated transfer-line to the infrared detector, as shown in Figure 1 below.

However, thermoanalytical techniques are actually preferred, since they allow the enhancement and quantification of each single gaseous evolution process. A quali/quantitative information can be obtained by the mass loss recorded, and consequently, thermal analysis is the most frequently on-line-coupled system to a MS or IR spectrometer.

**Figure 2:**
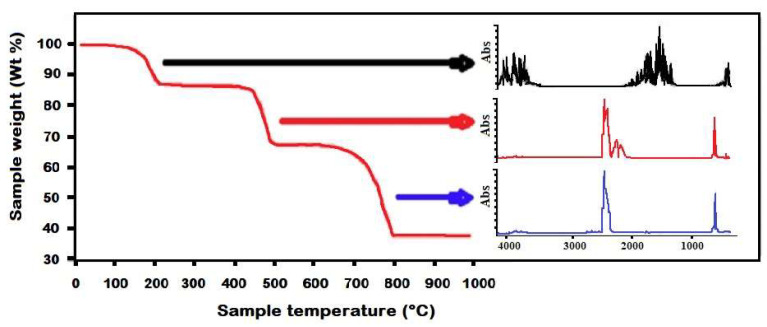
TG-FTIR analysis of a three-step decomposition example.

In Figure 2 a typical example is shown.

Following the recently published review on EGA-MS applications [12], in this review, the very recent analytical applications of FTIR-evolved gas analysis, selected among those published in 2019–2021 and early 2022, are collected and briefly described, to resume useful starting references. The number of publications on hyphenated techniques continues to grow in the areas of specialized applications; as a consequence, it is not unusual for an article on the topic to appear in an unfamiliar journal or a trade-specific publication. The aim is to help researchers to easily find applications, which are sometimes difficult to locate.

## 2. Applications to Polymers

Reliable chemical identification of specific polymers in environmental samples represents a major challenge in plastic research, especially with the wide range of commercial polymers available, along with variable additive mixtures. The TG-FTIR-GC-MS on-line system offers a unique characterization platform that provides both physical and chemical properties of the analyzed polymers. Nel et al. presented a library of 11 polymers generated using virgin plastics and post-consumer products. EGA was able to differentiate, and this library was then used to characterize real environmental samples of mesoplastics collected from beaches in the U.K. and South Africa [13].

Four isostructural lanthanide coordination polymers were hydrothermally synthesized and characterized by Rusinek et al. [14]. By TG-FTIR, it was possible to detect water, carbon oxides, m-xylene and methane as the main volatile products of complexes and thermal decomposition in nitrogen [14].

A sort of low-density polyethylene was characterized by several techniques, including EGA-FTIR, in the nitrogen flow. The applied kinetic scheme and corresponding kinetic parameters was suitable for the prediction of the thermal behavior of low density polyethylene in some temperature programs and can be used for the design of pyrolysis reactors necessary for the conversion of waste into low molecular mass chemicals, which can be used as raw materials for chemical and petrochemical industries [15].

Two new 3D coordination polymers were assembled from a virtually unexplored p-xylylene-bis(2-mercaptoacetic) acid linker. The FTIR coupling for EGA completed the characterization of the coordination polymers [16].

An innovative investigation on the thermal decomposition of the elastomer containing the fluoroolefin segment was carried out by a simultaneous DSC-TG-MS-FTIR to detect the thermal decomposition products [17,18].

The effect of heating rate and operating temperature on the product yield and composition as a consequence of the decomposition of polyamide via slow pyrolysis was reported by Pannase and coworkers [19]. The degradation markers and plasticizer loss of cellulose acetate films during ageing was described by Liu et al. [20].

Polyamide 66 is widely used in the polymer market, and there is a risk of dust explosion during processing and manufacturing. The explosion hazard of the PA66 dust and the inerting effect of melamine polyphosphate in PA66 dust were evaluated by testing the explosion characteristic parameters. The results demonstrated the maximum explosion pressure, minimum ignition energy and minimum ignition temperature. The EGA-FTIR results demonstrated that C_3_H_6_, produced during the pyrolysis of PA66, was the main combustible gas [21].

The catalytic effect of potassium additives on lignin pyrolysis was studied using two Cβ-O-type lignin-related polymers with a carbonyl group (C[dbnd]O polymer) and a hydroxyl group (OH polymer), respectively, on Cα. With or without potassium additives, the two polymers were analyzed by EGA-FTIR and Py-GC/MS [22].

Microplastic particles are currently detected in almost all environmental compartments. The results of the detection vary widely, as a multitude of very different methods are used with very different requirements for analytical validity. Goedecke and coworkers compared four thermoanalytical methods with their advantages and limitations, including EGA-FTIR-MS [23].

An investigation of the side chain effect on gas and water vapor transport properties of the anthracene–maleimide-based polymers of intrinsic microporosity was proposed by Caliskan et al. [24].

Pentamidine, an antiparasitic drug used for the treatment of visceral leishmaniasis, was modified by Scala et al. with terminal azide groups and conjugated to two different polymer backbones. This new hybrid polymer was proposed, respectively, as non-targeted and targeted drug delivery systems against Leishmania infections. Moreover, pentamidine was encapsulated into nanoparticles by the oil-in-water emulsion method, with the aim to compare the biological activity of the bioconjugates with that of the classical drug-loaded delivery system [25].

## 3. Applications to Complexes and Compounds

A cobalt (II) complex of diclofenax was synthesized and characterized by Gacki et al. [26] using several techniques, including TG-MS and TG-FTIR methods.

The oxidation behavior of six linear and branched alkanes was investigated by Yuan et al. to compare the similarities and differences between branched and linear alkanes. EGA-FTIR results indicated that both branched and linear alkanes demonstrate only low-temperature oxidation, which implies that alkanes barely contribute in coke formation and thus, no fuel was deposited [27].

Zapala and coworkers compared the spectral and thermal properties and antibacterial activity of new binary and ternary complexes of Sm(III), Eu(III) and Gd(III) ions with N-phenylanthranilic acid and 1,10-phenanthroline [28]. Świderski et al. studied the structural, spectroscopic, theoretical and thermal properties of metal complexes of pyridazine-3-carboxylic acid and pyridazine-4-carboxylic acids with Zn(II), Mn(II), Cu(II), Ni(II) and Co(II) [29]. Ferec et al. studied new complexes of Mn(II), Co(II), Ni(II), Cu(II) and Zn(II) with the ligand formed by the condensation reaction of isatin with glutamic acid [30]. New complexes of Mn(II), Fe(III), Co(II), Ni(II), Cu(II) and Zn(II) ions with niflumic acid were obtained in a solid state using a green synthesis method, and by the use of the coupled TG–FTIR–MS techniques, the decomposition pathways of the synthesized compounds were examined and the gaseous products released during thermal oxidation and pyrolysis of the complexes were identified [31].

The behavior of four diethyl hydrazine-yl-dienebutanedioates, which act as anticancer agents, was studied by Worzakowska et al. using the simultaneous TG/DSC-FTIR-QMS evolved gas analysis [32].

Effect of N,N′-dimethylformamide solvent on structure and thermal properties of lanthanide(III) complexes with flexible biphenyl-4,4′-dioxydiacetic acid was reported by Głuchowska et al. [33].

Rasi et al. [34] reported the effect of triethanolamine on the pyrolysis of the single-salt propionate-based solutions.

Heterometallic di- and trinuclear copper lanthanide complexes with an alcohol-functionalized compartmental Schiff base ligand were synthesized by Cristóvão and coworkers and characterized by EGA-FTIR [35]. Biomimetic complexes with transition metal ions were extensively investigated by Risoluti and Materazzi; on the basis of the results of a ten-year-long study on dicarboxylic [36], methylimidazole [37], pyridylimidazole [38], aminoimidazole [39,40], aminomethylimidazole and aminomethylbenzimidazole [41,42,43], imidazoledicarboxylic [44,45,46] and Schiff Base ligands [47,48], a suggested systematic decomposition model was proposed [49].

As a new solvent with a broad prospect, ionic liquids are widely used in the catalysis, organic synthesis, and electrochemistry due to their peculiar physical and chemical properties. Nonetheless, their safety issues are often overlooked. Wang et al. decoded the thermal reaction and decomposition hazard characteristics of ionic liquids. To predict the mechanism of decomposition in the early stage, the products were analyzed by TG-FTIR, and a huge amount of toxic gases were detected in its decomposition products, which are mainly HCN and NH_3_ [50].

The development of microwave-assisted processes involving the use of deep eutectic solvents is a fast-expanding research topic. A family of deep eutectic solvents composed by choline chloride and two types of hydrogen bond donors (carboxylic acids or polyols) was prepared and its microwave heating response and thermal heating behavior were investigated as well as compared to that of the single components by EGA-FTIR [51].

Although some thermoanalytical studies regarding the thermal analysis of dipyrone sodium monohydrate had already been presented in literature, the results are incomplete when no evolved gas analysis has been presented. Medeiros et al. [52] revisited the thermal degradation of dipyrone sodium monohydrate regarding the evolved gases, which are intermediates produced during heating and inorganic residue.

Tert-butyl peroxy-2-ethyl hexanoate, an important organic peroxide, is widely used as a polymerization initiator and curing agent in the chemical industry. Its thermal instability due to the presence of the peroxide bond may incur a decomposition reaction and cause further thermal runaway. The pyrolysis characteristics were assessed by EGA-FTIR and GC/MS, and the most likely pyrolysis mechanism was proposed [53].

## 4. Applications to Metal–Organic Frameworks

The impact of different synthetic procedures on the structure and thermal properties of coordination polymers was determined by Vlasyuk and Łyszczek. The prepared complexes were fully investigated and the thermal stability of the produced complexes was correlated with the synthesis conditions. Additionally, based on the volatile products of metal complexes decomposition, the mechanism of their pyrolysis was proposed in relation to their structures [54].

Bifunctionalized mesoporous silica materials were prepared by the sol–gel method applying the newly proposed sequence of addition of the used silanols. The CO_2_ adsorption properties of the synthesized bi-functionalized hybrids were investigated. The presence of isocyanurate groups in the hybrid silica framework significantly improved the textural characteristics and sorption capacities [55]. Hierarchically porous metal–organic frameworks with micropores, mesopores and macropores have been successfully fabricated by using compressed CO_2_. The possible mechanism of formation was proposed according to the results of the experiments [56].

The effect of two-dimensional zeolitic imidazolate frameworks-L on flame retardant property of thermoplastic polyurethane elastomers was reported by Xu and coworkers [57].

A series of 3D metal–organic frameworks as the organic linker and various lanthanides were prepared and studied by Dascalu and coworkers. TG-FTIR analysis of the resulting coordination polymers, as well as the powder X-ray diffraction measurements, demonstrated that compounds containing La^3+^, Nd^3+^ and Gd^3+^ possess only the emission associated with the anion of the organic ligand, while networks containing Dy^3+^ and Sm3^+^ display dual metal- and ligand-centered luminescence, with the organic linker acting as a good antenna for Tb^3+^, Eu^3+^, Dy^3+^ and Sm^3+^ ions [58].

Two novel heterobimetallic metal–organic frameworks were prepared via the solvothermal method, and their structures and composition were characterized. The thermal decomposition characteristics and their catalytic performances were also studied by the coupled FTIR-MS-EGA. The thermolysis catalytic mechanisms were studied by analyzing the transformation of gas products during the pyrolysis of mixtures [59].

## 5. Applications to Catalyst

The catalytic pyrolysis process is an environmentally friendly technology used to decompose the organic waste into chemical and volatile compounds with high abundance and quality. Yousef et al. decomposed the organic fraction of the end-of-life glass fiber-reinforced epoxy resin composites over ZSM-5 zeolite catalyst into energy products, thus liberating a fiberglass fraction [60].

Transition metal phosphides are promising materials for catalysis and their synthesis procedures commonly require costly or hazardous reagents. Material characterization can be confirmed by several techniques. Tong and coworkers determined the in situ generated reducing gases (CO, H_2_, PH_3_, etc.), detected by TG-FTIR [61].

The pyrolysis process has been adapted as a sustainable strategy to convert metallized food packaging plastics waste into energy products (paraffin wax, biogas, and carbon black particles) and to recover aluminum. Using the TG-FTIR-GC–MS system, Eimontas and coworkers observed and analyzed the thermal and chemical degradation of the obtained volatile compounds at maximum decomposition peaks [62].

Catalytic pyrolysis of popular sawdust in upgrading bio-oils as renewable energy has been studied by Li et al. via Fe-modified hierarchical ZSM-5. The results indicated that alkali treatment and Fe loading of the catalyst introduced a hierarchical and porous structure and improved its acidity, leading to high mono-aromatics and olefins selectivity. The on-line EGA-FTIR characterization demonstrated the volatiles’ release [63].

Pyrolysis plays a critical role in clean coal technology and energy conversion and is also conducive to the early peaking of carbon dioxide emissions. Light tar production from traditional coal pyrolysis using various iron-based catalysts for chemicals and fuel oil has gained attention recently. Song et al. initially used waste hematite for the catalytic upgrading of rapid coal pyrolysis. Subsequently, on-line EGA-FTIR and Py-GC-MS were utilized to compare the performances, kinetic parameters and volatile composition of coal pyrolysis with Fe_2_O_3_ and hematite. The results demonstrated that the maximum weight loss rate of coal pyrolysis increased with this addition, indicating that the pyrolysis reaction became more rapid and intense with iron-based catalysts [64].

Parameters controlling the reduction of nickel hydrotalcite-based catalysts have been investigated by Muller et al. in order to optimize the activity of the catalyst for CO_2_ methanation [65].

Three high-energy multicore ferrocene-based catalysts containing energy bonds were designed and synthesized to reduce the high temperature decomposition temperature and increase the heat release of ammonium perchlorate as well as improve anti-migration performance for the development of composite solid propellants. The thermal decomposition gaseous products under the catalysis was confirmed by EGA-FTIR to explore the catalytic decomposition mechanism [66].

Catalytic fast co-pyrolysis of biomass and plastic is an effective method to achieve high-quality bio-oil production. Shi et al. described catalysts with different Ni loadings, prepared and characterized in detail by using a variety of advanced analytical techniques. The effects on the catalytic performance were analyzed by microPy-GC/MS and EGA-FTIR methods. The results demonstrated that an appropriate amount of the Ni addition can effectively modulate the physicochemical properties [67].

## 6. Applications to Flame Retardants

Nylon is one of the most important engineering polymers used in textile, electronics and automotive applications. In addition to good mechanical properties, flame retardancy is desirable in these applications. Nylon is typically processed at high temperatures, and very few non-toxic synthetic flame-retardant additives are stable at these temperatures. Xia et al. reported the use of chemically modified Tannic acid—a naturally occurring polyphenol—as a more stable char forming flame retardant additive for Nylon. The evolved gas analysis through TGA-FTIR revealed that, in the presence of Tannic acid, some of the primary amide/amine and secondary amide species in Nylon 6 degrade into less flammable moieties such as ammonia, while aromatic moieties enhance char formation in the condensed phase, thus reducing flammability [68].

The thermal and fire behaviors of a high-performance polymeric material–polyether ether ketone (PEEK) was investigated by Ramgobin and coworkers. The TGA-FTIR-evolved gas analysis demonstrated that the initial decomposition step of the material may lead to the release of noncombustible gases and the formation of a highly crosslinked graphite-like carbonaceous structure. Based on the fire behavior and the identification of pyrolysis gases evolved during the decomposition of PEEK, the enhanced fire resistance of PEEK was assigned to the dilution of the flammable decomposition gases as well as the formation of a protective graphite-like charred structure during its decomposition [69].

A systematic series of flexible polyurethane foams, with different concentrations of flame retardants, was prepared by Chan et al. to test an approach to enhance flame retardancy and smoke suppression The pyrolysis behaviors and the evolved gas analysis were conducted by TG-FTIR, and highlighted the greater char yield due to the synergistic effect than the sum of individual effects [70].

The polystyrene composite containing the self-expanded intumescent flame retardant (polyphosphate ammonium and expandable graphite) was blended with three butyltriphenylphosphine-based chelate borates, to evaluate their effect on flame retardancy. The pyrolysis gaseous products were investigated by EGA-FTIR [71]. Several different Polyurethanes were prepared from the solvent and catalyst free reaction mixture containing polyethylene glycol, 4,4′-methylene diphenyl diisocyanate and 1,3-bis(2-hydroxyethoxy) benzene and/or castor oil. The probable degradation mechanism to study the role of β-hydrogen on the thermal stability was proposed and discussed based on the EGA-FTIR results [72].

Biomass phytic acid has a potential flame retardant value as the main form of phosphorus in plant seeds. Phytate-based flame retardants were synthesized, characterized and introduced into rigid polyurethane foam as flame retardants by a one-step water-blown method. EGA-FTIR enhanced the release intensity of flammable gases (hydrocarbons and esters) and toxic gases (isocyanate, CO, aromatic compounds and HCN) of composites, which was significantly reduced after the addition of PA-Fe [73].

It is important to develop halogen-free flame retardants for epoxy resin to reduce the fire risks. Cyclotriphosphazene-based derivatives have been proved to be effective phosphorous-containing flame retardants. Ning and coworkers demonstrated the preparation and application of a novel aminothiazole-based cyclotriphosphazene. TG-FTIR and pyrolysis gas chromatography/mass spectrometry were used to analyze chars and pyrolysis behaviors demonstrating a high efficiency for reducing fire hazards through a bi-phase mechanism [74]. Novel flame retardants were synthesized for treatment of cotton fabrics. TG-FTIR analyses indicated that these flame retardants change the thermal decomposition pathway of cellulose, and the combustible gases generated at high temperature are much lower than that of pure cotton. Flame retardant treated cotton fabric had excellent flame retardancy and durability, with whiteness and breaking strength being well maintained [75,76,77,78,79,80].

Luo et al. proposed an organic/inorganic phosphorus–nitrogen–silicon flame retardant, synthesized by the Kabachnik-Fields reaction and the sol-gel method, then used as a reactive flame retardant to prepare flame-retardant and smoke-suppressant epoxy resins. Evolved gases and char residues were studied by TG-FTIR, the results certified the charring effect of the phosphaphenanthrene group and the enhancing effect of the silicon group. Subsequently, the flame inhibition effect and lesser combustible gases release enhanced the flame-retardant properties [81]. Effects of a reactive phosphorus–sulfur containing a flame-retardant monomer on the flame retardancy and thermal and mechanical properties of unsaturated polyester resin were reported by Dai and coworkers [82].

Commercial flame-retardant rubber samples were investigated by EGA–FTIR and a fire propagation apparatus under different external heat fluxes to study thermal and fire characteristics. The characterization of the thermal decomposition, ignition time, heat release rate and limiting oxygen concentration could be used to evaluate the fire risk of flame-retardant rubber for the high-speed train department [83]. A new type of phosphorus- and nitrogen-containing flame-retardant coating was prepared, and a phosphorus-nitrogen cyclic polysiloxane flame retardant was synthesized by Zhang et al. It is found that both can enhance the flame retardancy of cotton fabrics, and EGA-FTIR indicated that treated cotton fabrics effectively exert a flame-retardant effect in the condensed and gas phases during combustion [84,85].

Based on the biodegradable material-polyethylene glycol as the plasticizer, with oxidized wood floor as the charring agent for polylactide, a series of flame-retardant biocomposites were prepared. EGA-FTIR allowed us to understand the flame-retardant mechanism, proving that the oxidized wood floor could be a new functional filler for polymer composites to further improve their flame retardancy [86].

Starch, distarch phosphate and hydroxypropyl distarch phosphate were used as “green” carbon sources with ammonium polyphosphate to prepare the flame-retardant reconstituted tobacco sheet by a paper-making process, and the effect of these starch-based flame retardants on their thermal degradation and combustion properties was preliminarily investigated and reported by Shao and coworkers. EGA by FTIR and MS coupling indicated that the starch-based flame-retardant coating promoted the char formation, inhibited the char combustion and the release of gaseous products [87,88,89,90].

The effect of aluminum on the flame retardancy was investigated by several authors [91,92,93,94,95,96].

Flame-retardant properties were also reported for an intumescent epoxy resin containing cyclotriphosphazene [97], for a polyacrylonitrile fiber modified with hydrazine hydrate and copper ions [98], for an montmorillonite added with a melamine quaternary ammonium salt [99] and for polylactic acid-conjugated composites [100].

## 7. Applications to Epoxy Resins

Mustata and coworkers studied the obtaining, characterization and thermal and fungal behavior of a green thermoset based on epoxidized soybean oil, castor oil maleic anhydride adduct and methyl nadic anhydride. EGA by FT-IR-MS coupling was used to establish the crosslinking behavior, evolved gases analysis and thermal decomposition mechanisms associated with their corresponding kinetic parameters. The main identified gaseous fragments were water, carbon dioxide, saturated and unsaturated hydrocarbons and carboxylic derivatives [101].

The effect of additives such as melamine, silica nanoparticles and a phosphorus-based compounds on the fire and mechanical performance of a bisphenol A diglycidyl ether (DGEBA)-based epoxy resin cured with isophoronediamine was investigated. The on-line EGA was used to propose the combined mode of action in the fire performance improvement of the epoxy system [102].

Battig and coworkers synthesized multifunctional P-based hyperbranched polymeric flame retardants with varying oxygen-to-nitrogen content. Using EGA-FTIR, Py-GC-MS, hot stage FTIR, and fire testing via cone calorimetry, detailed decomposition mechanisms were proposed [103].

A vanillin-derived cyclotriphosphazene-cored triazole compound was synthesized as a flame retardant for epoxy thermosets with high efficiency. To further study the flame-retardant mechanism, pyrolytic gas species were detected by TG-FTIR evolved gas analysis. The phosphazene groups clearly stimulated the production of an intumescent, compact and strong char layer, which improved the flame retardancy [104].

Liquid oxygen compatibility is the key to evaluating whether carbon fiber-reinforced epoxy composites can be used in a liquid oxygen environment. According to EGA-FTIR results, the carbon fiber composites demonstrated a better liquid oxygen compatibility than epoxy resin matrix [105].

A bio-based flame retardant was synthesized through a facile one-pot reaction with protocatechualdehyde and ethanol and characterized. The TG-FTIR analyses were performed and the results demonstrated that the synergistic effect on the flame retardant was ascribed to the protective effect of the denser char layer, the release of some phosphorus containing free radicals and the dilution effect of released nonflammable gases, such as H_2_O and CO_2_ [106].

The impacts of multi-element flame retardants on flame retardancy, thermal stability and pyrolysis behavior of epoxy resin was discussed by Zhao et al. [107]; meanwhile, the synergistic flame-retardant effect of epoxy resin combined with phenethyl-bridged DOPO derivative and graphene nanosheets was reported by Yan and coworkers [108].

The thermal behavior and smoke characteristics of glass/epoxy laminate and its foam core sandwich composite were comprehensively investigated using thermogravimetric analysis, TG–FTIR analysis, smoke density test and cone calorimeter test by Xu et al. [109].

Battig and coworkers synthesized a library of phosphorus-based flame retardants (phosphates and phosphoramides of low and high molar mass) and investigated their behavior in two epoxy resins (one aliphatic and one aromatic). The pyrolytic and burning behavior of the two resins were analyzed and compared to the results of the flame retardant-containing composites [110,111].

The synthesis and the thermal characterization of some hardeners for epoxy resins based on castor oil and cyclic anhydrides were described by Mustafa and Tudorachi. The main evolved gases during the degradation were identified from the FT-IR and MS spectra, demonstrating that the gaseous mixture contained water, carbon dioxide, some saturated and unsaturated hydrocarbons moieties, aromatic compounds and carboxylic derivatives [112].

## 8. Applications to Biomass

Catalytic fast pyrolysis is a prospective and effective route to produce value-added products from rice husk. Understanding the volatile compositions and its formation pathways as well as kinetics during rice husk pyrolysis is crucial and fundamental to regulate the quality of the target products, since catalysts interact directly with primary volatiles. Tian and coworkers employed TG-FTIR to conduct comprehensive experiments and demonstrated that in the three mass loss and gaseous product evolution stages, 2,3-dihydro-benzofuran was the major product for hemicellulose, while 2-methoxy-4-(1-propenyl)-phenol was potentially a key active intermediate, and was very unstable during the pyrolysis of the lignin constituent of rice husk [113]. Ding et al. reported the pyrolysis characteristics of the Jatropha curcas shell, demonstrating the change of the functional groups in organic compounds with temperature [114].

Bio-oil produced from the pyrolysis of sugarcane bagasse has the potential to be used as a sustainable and renewable energy source. Ordonez-Loza and coworkers investigated the pyrolysis (in nitrogen atmosphere) and combustion (in air atmosphere) of bio-oil from the sugarcane bagasse. The evolved gases in the TG furnace were carried to a FTIR cell, where the composition of the gases was analyzed [115].

The most important challenge in solid feedstock thermal conversion methods is minimizing CO_2_ emissions. The gasification of pine sawdust was investigated by Gao and coworkers for the reduction of CO_2_ by a calcium oxide loop. The results of the gasification process demonstrated that this procedure can be a possible solution for achieving negative carbon emissions [116].

Liu et al. characterized the catalytic combustions and emissions of litchi peels as a function of five catalysts as well as the effect of the best catalyst on the pyrolysis by-products. Litchi peels appeared to be a promising biowaste, with MgCO_3_ as the optimal catalytic option in terms of the bioenergy performance and emission reduction [117].

State-of-the-art reviews were published on the TG-FTIR approach to monitor the thermochemical conversion of the biomass for biofuel production by Ong and coworkers [118] and on the biomass pyrolysis technologies for value-added products by Amenaghawon et al. [119].

Rising demands for bioenergy and green products have led researchers to explore alternative, sustainable, and cost-effective feedstocks for biorefinery, among which perennial grasses adapted to marginal lands offer cost-effective and sustainable biomass to produce bioenergy and biochemicals without creating any competition with the agricultural lands. Yasmin and coworkers investigated the biorefinery potential of a fast-growing perennial grass Typha domingensis. EGA by FTIR-GC–MS confirmed the presence of alkanes, alcohols, organic acids, esters, ketones, aldehydes, amides and amines. These data indicated that T. domingensis has a substantial potential to become a feedstock of a sustainable biorefinery to produce bioenergy and biochemicals [120]. A comparative study on the two-step pyrolysis of different lignocellulosic biomass was proposed by Zhang and coworkers [121].

Tahir and coworkers studied soybean straw as a promising source of glycolaldehyde-rich bio-oil production and extraction. By EGA-FTIR, the highest glycolaldehyde concentration of 8.57% was obtained at 500 °C without the catalyst, while 12.76 and 13.56% were obtained with the catalyst at 500 °C [122]. Citrus aurantium is a widespread tree in the Mediterranean area, and it is mainly used as rootstock for other citruses. Aiello and coworkers reported a vacuum infiltration centrifugation procedure to isolate proteins from leaves. The results of profiling, combined with the top-down proteomics approach, allowed the identification of 78 proteins [123]. By EGA-FTIR analysis, Alves et al. demonstrated that the evolved pyrolysis products from cupuassu shells were dominated by alcohols, aldehydes, ketones, organic acids and aromatic/aliphatic hydrocarbons, supporting the utility of the cupuassu shell as a potential source for bio-based chemicals’ production. This study validated the cupuassu shell as a prospective feedstock for producing bioenergy and bio-based chemicals, also bringing useful information for engineering purposes in the design or simulation of large-scale pyrolysis reactors [124].

The effect of potassium on the pyrolysis of biomass components, by the study of the pyrolysis behaviors, product distribution and kinetic characteristics, was reported by Fan and coworkers [125].

## 9. Applications to Oil and Bitumen

In the search of new bioproducts, which could substitute petroleum derivatives and with the capability to attend the industrial demand, Bezerra et al. reported the synthesis of a babassu-oil-based biolubricant, its characterization and the impact in mineral lubricant properties when the biolubricant was used as additive. The gaseous products evolved during the process were analyzed by infrared spectroscopy, and the volatilization and combustion process could be described [126].

The chemical study of bitumen from stone tools from Italian Neolithic sites was carried out using analytical pyrolysis-based techniques, EGA-MS, EGA-FTIR and DSPy-GC/MS. The study was mainly aimed at demonstrating the suitability of analytical pyrolysis for studying archaeological bitumen and for obtaining information regarding its origin [127].

Abduhani and coworkers presented a comprehensive study on the characteristics and kinetics of the gas component releasing during oil shale pyrolysis. A TG-FTIR system was used to determine the decomposition regions and demonstrated that the evolved temperature and time span of each gas product are different. The results indicated that the control step gradually transits from the surface chemical reaction control to the joint control of reaction and diffusion with increasing temperature [128].

The characterization and dynamics of residual organics in oil sands’ fluid fine tailings was reported by Sasar and coworkers [129].

The pyrolysis characteristics of oil shale semi-coke and its extracted bitumen were described by Ding et al. [130,131].

## 10. Applications to Cellulose, Lignin and Lignite

Cross-linked poplar lignin with higher carbon content and 15 times the weight-average molecular weight was formed by doping boron phosphate in in situ composites, which was blended with poly(acrylonitrile-co-vinyl acetate) to prepare a low-cost partially bio-based composite. Shi and coworkers confirmed by TG-FTIR the acceleration in forming conjugated ladder structures and proposed the possible mechanism during the thermal stabilization, enhancing the great potential in developing low-cost carbon fibers [132].

The thermal stabilities of humic acids are critical for its effective utilization and relate closely with raw lignites. Li et al. studied the relationship between lignite and its derived humic acid by TG-FTIR [133].

The catalytic effects of ammonium dihydrogen phosphate on the biomass pyrolysis and its mechanism for the selective production of furfural and levoglucosenone were investigated by Li et al. The EGA results demonstrated that the decomposition of cellulose occur at a lower temperature and greatly enhanced the release of C-O groups and carbohydrates below 300 °C, indicating the breakage of the glycosidic bond and dehydration reaction during pyrolysis promoted by ammonium dihydrogen phosphate [134].

The thermal decomposition of exhausted sugar beet pulp and the resulting evolved gases were examined by TG-FTIR to identify the biomass fiber components. Lignin was more difficult to decompose, since its mass loss occurred over a broad temperature range (200–800 °C). The FTIR spectra obtained at these temperatures were consistent with the formation of large amounts of gases. Based on these results, the proposed approach allows lignocellulosic components in exhausted sugar beet pulp biomass to be efficiently screened [135].

Liang et al. applied a self-designed tubular reactor to observe the appearance of alteration and chemical structure evolution in hemicellulose pyrolysis during the whole xylan pyrolysis process [136].

Chen et al. constructed a phytic acid–silica hybrid system in wood by a vacuum-pressure impregnation process to improve its flame retardancy and smoke suppression. The mechanism underlying flame retardancy was analyzed by EGA-FTIR, and the results proved that the improved flame retardancy and smoke-suppression property of the wood are mainly attributed to the formation of an intact and coherent char residue with crosslinked structures, which can protect against the transfer of heat and mass (flammable gases and smoke) during burning. Moreover, the hybrid system did not significantly alter the mechanical properties of wood, such as compressive strength and hardness [137].

A well-known problem of the acrylic pressure-sensitive adhesives is that conventional flame retardants, while imparting good flame-retardant properties, compromise the cohesion and result in the unsatisfactory shear and peel strengths. Flame-retarded adhesives with improved shear and peel strengths were successfully prepared, and the TG-FTIR results confirmed that the flame-retardant behaviors occurred in the vapor phase [138]. The grass and celluloses’ initial stage of degradation under inert and oxidative atmosphere were reported by Zhao et al. [139,140].

Aiming to investigate the synergistic effect of K+ and anions on the primary products of its pyrolysis, microcrystalline cellulose was impregnated with KHCO_3_, KCl, K_2_CO_3_ and KOH. EGA by GC/MS and FTIR was used to investigate the volatiles during pyrolysis and catalytic pyrolysis, as well as the product distribution of pyrolytic bio-oil obtained from the fixed bed reactor, respectively [141].

The evolution of volatiles and kinetics, elucidation of reaction pathways, and characterization of gas, biochar and bio-oil were proposed by Chen et al. to provide insight into the biomass pyrolysis mechanism based on cellulose, hemicellulose and lignin [142].

An investigation on the Cunninghamia Lanceolate Cedar wood pyrolysis by TG-FTIR was reported by Chen et al. [143].

## 11. Applications to Materials and Composites

Analytical testing methods such as TG-FTIR and GC-MS were used by Li et al. to analyze the flue gas generated after the pyrolysis of an organic fireproof plugging material. The TG-FTIR analysis proved that carbon dioxide was the major component of the flue gas produced by pyrolysis, no matter whether it was under aerobic or restricted oxygen conditions. The safety assessment of the gases may adversely affect the fire rescue work, and the surrounding environment was carried out to reveal the potential hazards that may be exposed by using this organic fireproof plugging material in a fire [144].

Agarwood is a resinous wood of the Aquilaria species and has been used for various applications. Burning agarwood incense is a common practice in temples and homes in Asia. Kynam is widely regarded as a high-quality agarwood in the market. Recently, cultivated grafting Kynam has emerged as a new agarwood product in the market, which greatly affects the price of the high grading Kynam agarwood. Chen et al. investigated and compared the morphology, ethanol extract content and incense chemical profile. The incense smoke was analyzed by TG-FTIR and headspace gas chromatography–mass spectrometry. The results demonstrated that the heating of most incenses occurred below 200 °C, and HS-GC–MS analysis demonstrated the chemical compounds of incense smoke [145].

Common “glanded” cottonseeds contain the toxic compound gossypol that restricts the human consumption of the derived products. The “glandless” cottonseeds of new cotton varieties, in contrast, show a trace gossypol content, indicating the great potential of cottonseed for agro-food applications. He and coworkers comparatively evaluated the chemical composition of the two types of cottonseed kernels. Only the TG-FTIR analysis revealed apparent spectral differences between raw and extracted cottonseed kernel samples, indicating that some components were more susceptible to thermal decomposition [146].

In order to promote the resin modifying agent applied into pavement materials, a type of resin modifying agent was selected as the modifier to prepare modified asphalt. The thermal properties and functional groups of PA6 were studied by EGA-FTIR tests [147].

A DSC-TG-FTIR-MS comparative thermal research on energetic molecular perovskite structures was reported by Zhou et al. [148].

A three-dimensional carbon aerogel was prepared from waste corrugated cardboard by the procedure of slurrying, solvent replacement, drying and carbonization, in turn, and the product was explored as an all-in-one evaporator for solar steam generation without bulk water. Carbonization of the precursor was investigated using the thermogravimetric analyzer coupled with the Fourier-transform infrared spectrometer. The results demonstrated that CO_2_, CO, furfural and levoglucosan were released during pyrolysis, while the polymerization of newly formed char mainly resulted in the formation of CO_2_ and CO [149,150,151].

A new graphene oxide-functionalized coordination polymer composite was synthesized and characterized through SEM, EDS and XPS analysis as well as the TG-FTIR and TG-MS-evolved gas analysis, indicating its promising application as a combustion catalyst [152].

Polybenzoxazine composites, based on the phosphorous-containing bio-based furfurylamine type benzoxazines and bisphenol-A type benzoxazines, were developed for flame retardation. The fire-resistant mechanism was supported by the SEM-EDS and TG-FTIR-evolved gas analysis, proving composites as promising fire-retardant polymers [153].

In order to promote the resin modifying agent applied into pavement materials, a type of resin modifying agent was selected as the modifier to prepare modified asphalt. The thermal properties and functional groups were characterized by EGA-FTIR to confirm applying the feasibility in asphalt [147].

Precursor impregnation and pyrolysis is an important process to fabricate the fiber-reinforced SiC matrix composites, and a low viscous, curable, long-storable and affordable liquid precursor is urgently needed. To meet the above requirements, Yuan et al. proposed a liquid two-component precursor system. The effect of different crosslinkers on curing and pyrolysis process was investigated by EGA-FTIR-MS. Based on the results of the total ceramic yield, the optimized crosslinker of four candidate organosilicon compounds was proposed [154].

Bamboo charcoal and aluminum hypophosphite, both singly and in combination, were investigated as flame-retardant fillers for polylactic acid. A set of composites were prepared by melt-blending and tested for thermal and flame-retardancy properties [155].

An innovative hybrid nanocomposite of Ni–Al layered double hydroxide and carbon nanotubes was assembled via one-pot of the hydrothermally assisted coprecipitation approach and was applied as the thermal stabilizer in the polyvinyl chloride resin. EGA-FTIR proved the increased maximum degradation temperature and the improved removal efficiency of hydrogen chloride [156]. Fire-retarded polymer nanocomposites based on polypropylene with clay and allylamine polyphosphate were designed and prepared by melt mixing. Significant enhancements in the fire safety performance were also observed by TG-FTIR as well as smoke and toxic gases [157].

Composite filaments comprised of lignin and cellulose nanofibers were fabricated by a microfluidic spinning technique together with the in situ interfacial complexation. After stabilization and carbonization, bio-based carbon fibers with fine graphite microcrystals were obtained and carbon lattice was highly oriented along the fiber direction, contributing to the superior macro-performance. A combined TG-FTIR approach was applied to further analyze the carbonization process [158].

Using phytic acid and piperazine as raw materials, a series of rigid polyurethane foam/piperazine phytate composites were prepared by a one-step water-blown technology. EGA-FTIR indicated that phytic acid and piperazine significantly inhibited the release of toxic gases and flammable gases, effectively improving the fire performance of the composites [159].

Coupled DSC-TG-FTIR-MS were used by Guo et al. to study the mechanism of two typical binders to demonstrate that both can induce premature decomposition and increase the activation energy. It was found that the rapid mechanism of binder and active intermediate products inhibit the reaction of relatively inert intermediate products and prolong the continuous generation time of gas products in the composite particles, which delays the decomposition [160].

A series of rigid polyurethane foam/aluminum hypophosphite/expanded graphite composites were prepared by the one-step water-blown method. Based on the analyses (including EGA by FTIR), the flame-retardant mechanism of these new composites was proposed [161]. Fire-retardant polyurethane foam composites based on the steel slag/ammonium polyphosphate system were proposed by Yang et al., by a novel strategy for the re-utilization of metallurgical solid waste [162].

A facile preparation synthesis of magnetic porous carbon monolith from the waste corrugated cardboard box for solar steam generation and adsorption was reported by Ma and Cao [163].

## 12. Applications to Nanoparticles and Nanomaterials

Surface functionalization is a key factor for determining the performance of nanomaterials in a range of applications and their fate when released to the environment. Nevertheless, it is still relatively rare that surface groups or coatings are quantified using methods that have been carefully optimized and validated with a multi-method approach. Kunc and coworkers quantified the surface groups on a set of commercial ZnO nanoparticles modified with three different reagents ((3-aminopropyl)-triethoxysilane, caprylsilane and stearic acid). The TG-FTIR evolved gas analysis, quantitative solution NMR and X-ray photoelectron spectroscopy demonstrated that there are significant mass losses from the unmodified samples, which are attributed to surface carbonates or residual materials from the synthetic procedure used. These results highlight the importance of the developing reliable methods to detect and quantify surface functional groups and the importance of a multi-method approach [164].

Thermoanalytical methods, including TG-FTIR, TG-MS and TG-GC-MS, were used for the characterization of nanomaterials and nanocomposites to ascertain the influence of nanoparticles on the thermal stability and degradation mechanism of polymer nanocomposites. EGA methods helped to determine the thermal properties of nanoparticles and to understand the influence of nanomaterials on polymer phase transitions, thermally induced chemical reactions, as well as thermal transport properties in advanced polymer composites [165,166]. The employment of transition metal oxides as catalysts has been widely popularized in the thermal decomposition and combustion process, especially in propellants and explosives application areas. The focus of the study proposed by Zhang and coworkers was on investigating the catalytic effects of metal oxide nanoparticles on the thermal decomposition mechanism and kinetics behavior of one typical energetic material. The thermal decomposition mechanisms were deduced by TG-FTIR-MS analysis [167,168].

Functionalized carbon nanotubes, using two polyamine polymers, polyethyleneimine and polyamidoamine dendrimer, were investigated by EGA in order to address the preparation strategies to obtain low cytotoxic compounds with the ability to conjugate microRNAs and, at the same time, to transfect the endothelial cells efficiently [169].

Well-dispersed polyethylene nanocomposites were developed by Attia and coworkers. Montmorillonite as aluminosilicate clay layers was modified using organic silanes of different side chains. The developed organoclays were dispersed uniformly in the PE matrix producing well exfoliated and dispersed polymer nanocomposites. The toxicity of gases evolved during the combustion process of PE and their polymer nanocomposites was evaluated using EGA-FTIR [170].

Liu et al. proposed an improvement of the platinum nanoparticle-immobilized α-zirconium phosphate sheets on the tracking and erosion resistance of silicone rubber [171]. Nanoparticles were also involved to improve the heavy oil oxidation performances by oil-dispersed CoFe_2_O_4_ nanoparticles in the in situ combustion process for enhanced oil recovery [172].

## 13. Applications to Zeolites

The catalytic behaviors of Ca-modified HZSM-5 during the oil shale pyrolysis process were investigated in a tubular rector and by TG-MS-FTIR. The results demonstrated that the molecular sieve can significantly increase yields of C1−4 aliphatic hydrocarbons and reduce their evolution temperatures. After modification, Ca/HZSM-5 can reduce yields of CO_2_, increase yields of shale oil and decrease lengths of aliphatic chains in shale oil [173].

Liang et al. reported a facile and Ti-incorporation-improved liquid–solid impregnation method for Ti-β zeolite preparation by incorporating a unique Ti precursor into the defects of dealuminated β zeolite. The function mechanism of this unique Ti precursor was provided based on EGA characterization results, and it suggests that the Ti precursor with two types of substitution groups, which show different reactivity, would enhance the Ti incorporation into the zeolite framework [174].

Liu et al. investigated the effects of the zeolite 4A on the flame retardancy and thermal stability of aluminum β-(p-nitrobenzamide) ethyl methyl phosphinate in acrylonitrile–butadiene–styrene copolymer. The TG-FTIR analysis and morphology of the char residues from the scanning electron microscopy further demonstrate that zeolite 4A could retard the volatilization of hydrocarbons and promote the char formation in the combustion process of ABS [175].

Various zeolites (4A, Y, 13X) and related fillers (molecular sieves and coal fly ash) were used to prepare PLA composites and to assess their degrading effect on the polyester matrix under different processing conditions, at various zeolite/filler loadings. The degradation of PLA during the preparation and processing of PLA–zeolite composites, or due to the presence of fillers, was evidenced by specific analyses, including EGA-FTIR [176].

## 14. Applications to Clays

Mineralogical characterization of clays used in the manufacturing of traditional ceramic products is critical for guaranteeing the quality of the final product, but also for assessing the environmental impact of the industrial process in terms of atmospheric emissions. EGA-FTIR and EGA-MS provides more valuable information and lower limit quantification than other primary techniques, such as X-ray diffraction or simple infrared spectroscopy. Silvero et al. developed an analytical procedure using evolved gas analysis to identify and quantify minerals such as chlorides, sulfides, carbonaceous materials and minor clay minerals and to include the analysis of acid emissions during the ceramic firing treatment even if they are present at low quantitative levels [177]. Mo et al. studied the changes in mechanical properties of rocks at high temperatures, including quartz, plagioclase, amphibole and biotite. The TG-FTIR evolved gas analysis was performed to examine the physio-chemical alterations of minerals at high temperatures [178].

Pulidori and coworkers presented the results obtained from the thermal analysis of a set of geomaterials (clays, pyroclastic materials, and industrial recycled materials) to be used as raw materials for the synthesis of geopolymers, specifically designed for the conservation of Cultural Heritage buildings. The FTIR evolved gas analysis was useful to characterize their thermal behavior [179]. The early properties and microstructure evolution of alkali-activated brick powder geopolymers at varied curing humidity were reported by Shen et al. [180].

The catalytic effect of clay rocks as a matrix for heavy oil combustion in the in situ combustion process for enhanced oil recovery was investigated by EGA-FTIR together with kinetic calculation. The results revealed that three types of clays demonstrate a good catalytic effect verified by a significant reduction in activation energy, mainly manifested as merging the fuel deposition into high-temperature oxidation and shifting the temperature peak into lower temperature [181].

## 15. Applications to Coal

The modeling of pyrolysis can help to understand, predict and optimize many industrial processes. Ma et al. proposed an improved parallel reaction model, which tackles the issues of precision and rationality of the popular distributed activation energy model. The model was established by optimizing the parameters of sub-reactions, which were estimated via the TG-MS-FTIR data of a bituminous coal [182].

The spontaneous combustion behaviors of pulverized coal including anthracite, coking coal, nonstick coal and lignite were analyzed by the FTIR-evolved gas analysis. The correspondence between every key temperature point and coal characteristics was described in detail [183].

Chemical looping combustion has been well developed as a novel combustion technology for the simultaneous completion of the coal combustion and CO_2_ capture with a low energy penalty. Wang et al. prepared a CaSO_4_–CoO mixed oxygen carrier, and experiments based on TG-FTIR evaluated the reaction characteristics and evolution of the gaseous products [184].

The combustion behavior and gas product characteristics of coal gangue were studied by Bi et al. [185].

Li et al. characterized coal gangue and raw coal, since they can catch fire due to spontaneous combustion. The combustion characteristics, functional groups and kinetic behavior were investigated to assess their thermal stability characteristics [186].

Investigation into the effect of Fe_2_O_3_ on the combustion characteristics and kinetic of coal/char co-combustion were reported by Hu and coworkers, based on the FTIR-evolved gas analysis [187].

On-line-coupled EGA-FTIR was used to analyze the co-pyrolysis characteristics of the coal slime and coffee industry residues, mixed according to different mass ratios. Through the detection of gas emission and mass loss rate with temperature changing, the results demonstrated that the co-pyrolysis has a synergistic effect [188].

The petroleum ether-extracted residues of the low temperature coal tar were separated and analyzed by EGA-FTIR and Py-GC/MS. It was demonstrated that the pyrolysis volatiles change completely with the rising temperature [189].

## 16. Applications to Membranes

Wang and coworkers studied the separation of azeotropic mixtures such as methyl acetate and methanol via a membrane, since the membrane must be able to withstand these harsh solvents and provide good flux and selectivity. Evolved gas analysis revealed that the titanium components in TiPCS inhibit and/or reduce the densification of the network structures at elevated temperatures [190].

End-life ultrafiltration polymer nanocomposite membranes and their valorization into high added-value products were studied by Yousef and coworkers, using the pyrolysis treatment. EGA-FTIR and GC-MS coupled systems proved the volatile products released from the pyrolysis process of the membranes, indicating that end-life polymer membranes can be used as a new source for renewable energy [191].

## 17. Applications to Coatings

The thermochemical properties of a solvent-borne two-component polyurethane acrylate-based wood coating were studied using TGA-FTIR to determine the structural features of the cured coating material [192].

## 18. Applications to Environment and Health

Dioxins reduction is crucial for the pyrolysis of municipal solid waste and fundamental for environment and human health. A torrefaction-pyrolysis experiment was performed in a tubular furnace system, and the weight loss and chlorine release (mainly in the form of hydrogen chloride) were evaluated. The dioxins’ distribution in the products were detected by high-resolution mass spectrometry, and EGA-FTIR clarified the chlorination of PAHs [193].

Emission characteristics of nitrogen and sulfur containing pollutants during the pyrolysis of oily sludge with and without catalysis were studied and characterized by Liu et al. [194,195].

A novel multiway approach by spectroscopy and thermogravimetry associated with chemometrics was developed, providing a multiparametric characterization of vitreous humor as a function of the time since death [196,197]. β-Thalassemia is a hemoglobin genetic disorder characterized by the absence or reduced β-globin chain synthesis, one of the constituents of the adult hemoglobin tetramer. The possibility of using a thermoanalytical approach in combination with the rheology [198] or chemometrics as innovative tools for β-thalassemia detection was proposed [199,200,201,202,203,204,205,206,207]. Oral fluids’ rapid testing was also proposed by combining EGA and spectroscopy [208,209].

Thermal degradation and the combustion of pesticides lead to the emission of chemicals that are harmful and dangerous for humans and the environment. Boruckaet al. studied the thermal decomposition of triadimenol and analyze organic compounds, and in particular, the dioxins precursors released during that process [210].

Imbalance of the iron level in the body causes several diseases. In particular, the low level of iron during pregnancy is responsible for iron deficiency anemia, and even of neurodegenerative diseases. Catauro and coworkers developed a system able to release ferrous ions in a controlled manner and confirmed the Fe(II) presence in the silica matrix [211].

Ludovici et al. studied the skin surface lipids resulting from the blending of sebaceous and epidermal lipids, which are derived from the sebaceous gland secretion and the permeability barrier of the stratum corneum, respectively. Untargeted approaches demonstrated that the relative abundance of numerous lipid species was distinctive depending upon the different density. Results indicated that the secretion intervenes in shaping the lipid composition of the epidermal permeability barrier [212].

## 19. Applications to Sewage Sludge

Hazardous oil sludge poses a great challenge to the environment, whereas conventional treatment methods (i.e., incineration or pyrolysis-incineration) are relatively less value-added and will bring about air pollution problems. Catalytic co-pyrolysis with waste biomass to produce platform chemicals was studied using EGA-FTIR and Py-GC-MS methods by several authors. The results demonstrated that for the non-catalytic co-pyrolysis of rice husk and oil sludge, the main synergy on weight loss was the greatly lowered initial pyrolysis temperature of rice husk at the lower temperatures and the reduced weight loss ratio of oil sludge within the higher temperature range [213,214]; the dewatered sewage sludge was upgraded to hydrochar using hydrothermal conversion because of superior fuel quality and avoidance of energy-intensive dewatering [215]; Chinese liquor and bamboo biomasses were studied to assess the potential to produce clean energy and biochemicals via pyrolysis and co-pyrolysis [216]; EGA by simultaneous FTIR and MS was proposed by Lin et al. [217].

Xylan from corn cob and high-density polyethylene were investigated by an on-line TG-FTIR-MS system to explore the co-pyrolysis process, which demonstrated the blends to decompose more easily [218].

Biomass and sewage sludge were characterized under N_2_, CO_2_ and mixed atmospheres by Chen and coworkers [219].

The reducing gases produced and the NO reduction by sewage sludge combustion were investigated in a self-made cement precalciner by Xiao et al. The dual role of oxygen concentration in the production characteristics of reducing gases and the reduction efficiency of NO were evaluated experimentally. The coupled TG-FTIR analysis demonstrated the key reducing gaseous species produced by the sewage sludge combustion, and the experiments demonstrated that oxygen concentration had pronounced effects [220].

Du and coworkers demonstrated the effects of baking soda on the Co-hydrothermal carbonization of sewage sludge and Chlorella vulgaris and improved the environmental friendliness of the hydrochar incineration process [221].

## 20. Applications to Sulphur

Hierarchical porous carbons exhibited excellent performance for H_2_S removal with the sulfur capacity up to 426.2 mg/g at room temperature. The formation mechanism of the N-rich hierarchical porous carbons was investigated by EGA-FTIR, suggesting that CN pyrolysis result in hierarchical porous structure and rich N-containing functional groups. In addition, because of their high regeneration ability, they are promising catalysts to remove H_2_S efficiently with low cost and high reusability [222].

## 21. Applications to Tobacco

Investigation of the detailed pyrolysis characteristics of tobacco raw materials is important for the understanding of product design and consumption. Pyrolysis characteristics and kinetic models of cigar filler tobacco, cigar wrapper tobacco and flue-cured tobacco were investigated by EGA-FTIR. During the pyrolysis process, evolved gases including H_2_O, CO_2_, CH_4_, CO, carbonyls, alcohols, phenols and aromatic compounds were detected by FTIR [223].

Gu et al. investigated the effects of the hydrothermal temperature and residence time of hydrothermal carbonization on the properties of tobacco stems and their pyrolysis behaviors with catalysts [224].

To study the influences of the types and contents of aerosol carriers on pyrolysis and the smoke release of tobacco granules, the wet granulation of tobacco was prepared with different glycerin/propylene glycol ratios. The granule samples were studied by TG-FTIR), cone calorimeter and steady-state pyrolysis. The results demonstrated that with the increase in propylene glycol, the initial weight loss rate and total weight loss of tobacco granules increased significantly, and the infrared spectra indicated that tobacco granules began releasing aerosol carrier from 100 °C when propylene glycol was added [225,226].

## 22. Applications to Explosives and Propellants

Nitrogen-rich energetic materials have been widely studied because of their high thermal stability, insensitivity, excellent detonation performance and non-toxic characteristics. In particular, these compounds are well applied as gas-generating agents. As a nitrogen-rich heterocyclic framework, 1,2,4,5-tetrazine derivatives have demonstrated great potential. Guanidine salts of 3,6-bis-nitroguanyl-1,2,4,5-tetrazine, guanidine, aminoguanidine, diaminoguanidine and triaminoguanidine have been synthesized and characterized. Among the other applied characterization techniques [227], a simultaneous TG-FTIR-MS analysis of the thermal decomposition products revealed the main decomposition gaseous products being clean gases, without solid residue after burning, and consequently a potential green energetic material for gas-generating agents [228].

An overall decomposition mechanism of ammonium dinitramide in the gas phase was studied by Wang et al. by the TG-FTIR and TG-MS evolved gas analysis. Both calculated and experimental results indicated that the product N_2_O is first formed in the thermal decomposition of ADN(g). The systematic mechanistic investigations provided new and detailed insights on the mechanism and kinetics of the thermal decomposition of gaseous ammonium dinitramide, which is beneficial to other energetic material syntheses [229].

As a novel nitrogen-rich content material, 5-Amino-1H-Tetrazole has been considerably popularized in the propellants’ area recently. The pyrolysis is deemed as the prelude of combustion, which plays a crucial role in the combustion process; grasping the pyrolysis characteristics will be more beneficial to further explore the combustion properties of the 5-Amino-1H-Tetrazole-based propellant. The TG-FTIR technique was applied to capture the evolved signals of organic compounds to further deduce the most probable decomposition pathway [230].

As newly developed primary explosives, zinc carbohydrazide perchlorate and cadmium carbohydrazide perchlorate exhibited excellent performances, enhanced by a novel TG-FTIR-GC/MS simultaneous coupling [231].

Guo et al. reported the compatibility and thermal decomposition mechanism of the nitrocellulose/Cr_2_O_3_ nanoparticles studied using DSC and TG-FTIR [232].

Backdraft is an explosive fire phenomenon, which typically occurs during fire-fighting activities, occasionally leading to fire-fighter fatalities. Real backdraft incidents involve complex fuel gas mixtures consisting of the products of underventilated burning and pyrolysis following burnout. However, most experimental research into the backdraft has used methane gas or flammable liquids as fuel. Some aspects of real backdraft behavior have been studied by Wu et al. using the fire propagation apparatus with EGA-FTIR. It was concluded that the crucial factors determining whether backdraft occurs or not are the maximum temperature and the CO/CO_2_ ratio in the compartment, prior to opening the door [233].

A novel hydroxyl-terminated block copolymer binder was prepared through an in situ preparation method to replace the traditional HTPE binder. The thermal decomposition behaviors of the in situ-prepared HTPE binder and the traditional HTPE binder were investigated by EGA-FTIR, and the novel in situ-prepared HTPE binder exhibited a good thermal stability and superior energetic performance with great potential for applications in rocket propellant formulations [234].

Al/CuO nanothermites have displayed unique catalytic activities in accelerating the thermolysis of nitrocellulose and combustion characteristics of propellants depending on the morphology of CuO. TG-FTIR results revealed that CuO and Al/CuO play crucial roles in accelerating the dissociation of the –O–NO_2_ bond and the coacervate phase to change the pyrolysis mechanism of NC from an autocatalytic reaction to a modified n-th order reaction model [235].

Ferreira and coworkers reported an investigation on the thermal degradation of the biolubricant through EGA-FTIR and the characterization of the biodiesel as energetic raw material [236].

El-Sayed collected in a review the published papers on thermal decomposition, kinetics parameters and evolved gases during the pyrolysis of energetic materials using different techniques [237].

## 23. Applications to Waste Materials

Waste oil, such as waste cooking and acidic oil, are two promising carboxylic acids feedstocks that can be converted into hydrocarbons by the pyrolytic method. The TG-FTIR-MS on-line EGA results revealed that the pyrolysis conversion of waste oil was a direct and effective deoxygenation method, and the high alkene content endowing pyrolytic oil greatly demonstrated the potential to upgrade to a high-quality biofuel production [238,239].

Co-pyrolysis characteristics of kitchen waste with tire waste were studied by TG-FTIR and Py-GC/MS. The results demonstrated that a certain coupling synergistic interaction occurred between kitchen waste and tire waste and could be a potential way for improving the quality of pyrolysis oil [240].

Yousef and coworkers developed a new thermochemical strategy to extract butane from the billions of wasted COVID-19 masks generated. The experiments were conducted with 3-ply face masks over TG-FTIR and GC–MS measurements, and demonstrated that the decomposed samples are very rich in aromatic and aliphatic (-C-H) compounds. On these bases, the catalytic pyrolysis strategy over zeolite can be used effectively to dispose of COVID-19 masks and to convert them into a butanol compound that can be used as a liquid fuel and lubricant [241].

Waste tires, in addition to oily sludge, are severe problems in the environment. Co-pyrolysis features, synergistic effects and gas products were studied using EGA-FTIR. The results revealed that the oily sludge and waste tires’ co-pyrolysis has synergistic effects [242,243]. The co-pyrolysis of biomass and waste tires under the high-pressure two-stage fixed bed reactor was performed by Wang et al. [244] and by Kumar et al. [245]. Wang et al. investigated the hot char catalytic role in the pyrolysis of waste tires in a two-step process [246].

Non-tire rubber products (e.g., hoses, weather strips, vibration insulators and miscellaneous parts) are progressively increasing as scrapped and repaired vehicles rise, and are abundantly discarded due to various reasons, such as high-cost recycling, complex rubber components and poor mechanical properties. The non-tire rubber products have similar chemical components as scrap tires that have been widely applied in the field of road engineering, so they can be pretreated and used in road engineering as scrap tires. Sun and coworkers and Xia and coworkers studied asphalt binders modified with waste non-tire rubber with or without a microwave and/or waste engineering oil treatment by using the terminal blend process as crumb rubber. Many laboratory tests were conducted, including the TG-FTIR analysis, to test the storage stability, high-temperature performance, fatigue and other basic rheological properties [247,248]. Asphalt binders were the object of a study by Zhou et al., in which the combustion mechanism and gaseous products of asphalt binder at five different oxygen concentrations were studied by EGA-FTIR. The aim was to provide a more scientific basis for the analysis of asphalt combustion under low oxygen conditions in tunnel fires [249]. Advances in the detection of used drugs were also reported by the coupling thermal analysis and spectroscopy [250,251,252].

Singh and coworkers reported an investigation of polymers’ degradation, with particular focus on HDPE, PP, PS and PET individually and in mixed forms. Online TG-FTIR determined the functional groups present in the volatile fractions during single component pyrolysis and the interaction of polymers during co-pyrolysis [253].

Gautam and coworkers presented a thorough thermal analysis of the dead remains of mosquitoes, in order to unravel the composition of vapors evolved from the bug zappers decimating them. The deconstruction of proteins and fatty acids present in mosquitos was similar to that of microalgae in terms of the pyrolysis products [254]. Gan et al. studied the effects of dry and wet torrefaction pretreatment on microalgae pyrolysis, including the characterization analyzed by the EGA-FTIR and double-shot Py-GC/MS [255]. Yan et al. used the chemical looping gasification to study the effective utilization of microalgae’s energy [256,257]. EGA-FTIR and the kinetics of catalytic and non-catalytic pyrolysis of Microalgae Chlorella sp. Biomass were reported by Farooq et al. [258].

Niu and coworkers studied the effect of torrefaction on the evolution of carbon and nitrogen during the chemical looping gasification of rapeseed cake [259]. Niu and Shen detected the evolution of carbon and nitrogen during the chemical looping gasification of rapeseed cake with a Ca-Fe oxygen carrier [260].

The combustion of moxa floss was investigated by Zhang and coworkers. The results indicated that the combustion of moxa floss was a mild and slow process, releasing heat continuously and steadily, and consequently was an optimal material for thermal therapy and is beneficial for the development of an electric thermal stimulation alternative [261].

The chemical synthetic residual is one of the solid wastes generated from the pharmaceutical industry. The pyrolysis and combustion characteristics of the chemical synthesis residuals were investigated by Fang et al. using EGA-FTIR. The emission characteristics of combustion at low temperature were similar to that of the pyrolysis, while CO_2_ was found as a major gaseous product at high temperature [262].

With the rapid development of food waste anaerobic digestion, the safe treatment of its digestates is to be considered. The co-combustion of food waste anaerobic digestion with municipal solid waste provided a cleaner and sustainable solution, and the EGA-FTIR results demonstrated that it was beneficial to reduce the emissions of gaseous pollutants [263]. Torres et al. assessed the thermal transport properties, thermochemical characteristics and gaseous pollutants released during the co-combustion of different biomasses with oil shales [264]. Coal slime, coal gangue and raw coal mixtures were systematically investigated by Ma et al. [265] and Ni et al. [266,267]. The co-combustion of black liquor and oil sludge were explored by TG-FTIR to prove that the addition of oil sludge can promote the reaction in the early stage, as indicated by the combustion characteristic index and the flammability index [268].

Co-pyrolysis was extensively applied to several matrices: biomass and plastics as a promising method to alleviate environmental pollution and provide renewable energy [269]; bamboo residues for commercial development [270]; low-rank pulverized coal and direct liquefaction residue to produce a small amount of gases [271]; coal slime and cattle manure, to demonstrate that their mix will improve the pyrolysis performance [272]. Yin et al. studied the co–pyrolysis of de–alkalized lignin and coconut shell via pyrolysis, EGA–FTIR and machine learning methods [273]. The dynamic analyses of evolved syngas, bio-oils, biochars, interaction effects and reaction mechanisms of the co-pyrolysis of textile dyeing sludge and Pteris vittata (hyperaccumulator biomass) were characterized and quantified comparatively by Song and coworkers [274,275].

EGA by the coupled FTIR-MS was used to evaluate the pyrolysis of gaseous products and combustion performance of food waste digestate [276,277].

Chen and Chen investigated co-torrefaction followed by a co-combustion of intermediate waste epoxy resins and fir in the form of a mini-pellet to evaluate the potential of industrial wastes as biofuels for alternatives of coals. EGA-FTIR suggested that most of the torrefaction had a slight influence on the wastes due to their thermal resistance properties. Conversely, the fir was markedly affected by torrefaction, and the corresponding volatiles were the chemicals stripped or reacted from its components (hemicellulose, cellulose and lignin). Co-torrefaction was confirmed to help the quality of the biofuel from intermediate waste epoxy resins, being more homogeneous, and proved to be a promising route to transform waste epoxy resins into alternative fuels for industrial applications [278].

The combustion behavior, kinetics, thermodynamics and gas products of fine screenings classified from municipal solid waste were explored, indicating that the combustion process can be divided into four stages [279].

Pine waste was tested to calculate the yield of the desirable chemical compounds, and bio oil was produced at each corresponding temperature by Tahir et al. [280].

Both pyrolysis and incineration of waste vinyl panels, the main component of which is PVC, are difficult, complex and multifaceted processes, due to the occurrence of several physical and chemical phenomena. EGA-MS-FTIR was useful to identify the decomposition mechanisms of waste vinyl panels in the light of the circular economy [281].

To clarify the effect and mechanism of straw waste on the properties of asphalt materials, Qin and coworkers prepared two types of modified asphalt by using straw powder and incineration ash. The results indicated that ash has a greater potential in enhancing and improving the physical and thermal properties of asphalt vs. straw powder [282]. Dynamic pyrolysis behaviors, products and mechanisms of waste rubber and polyurethane bicycle tires were studied by Tang and coworkers [283].

Given the COVID-19 epidemic, the quantity of hazardous medical wastes has risen unprecedentedly. Xu et al. characterized and verified the pyrolysis mechanisms and volatiles products of medical mask belts, mask faces and infusion tubes via EGA-FTIR and Py-GC-MS analyses [284].

Evolution and distribution characteristics of fluorine during the incineration of fluorine-containing waste in a hazardous waste incinerator were reported by Li et al. [285].

Ma and Cao proposed a facile preparation of the magnetic porous carbon monolith from a waste-corrugated cardboard box for solar steam generation and adsorption [163].

Silicones are widely used in the medical and engineering field for their remarkable properties, but the resulting waste is an increasing economic and environmental problem. Pyrolysis can be a feasible way to recycle silicone waste. Qin et al. found anatase titanium dioxide to be very efficient in catalyzing the pyrolysis of silicones even at extremely low temperatures. TG-FTIR and Py/GC/MS analyses clarified the related catalytic mechanism and proposed it as a promising technology for inexpensive, highly efficient and sustainable features in the feedstock recycling of silicones [286].

## 24. Applications to Food

Chitins and Chitosans with different degrees of deacetylation were analyzed for the first time with TG-FTIR in order to evaluate the effect of deacetylation on the thermal decomposition process. The TG-FTIR results evidenced a complex gaseous mixture mainly composed of ammonia, acetic acid, acetamide, water, monoxide and carbon dioxide in proportions that are deeply dependent on the deacetylation [287].

Starch is widely used in industrial production and in every walk of life, and an increasing number of accidents of starch dust burning and explosions are occurring and have caused serious casualties and economic losses. To prevent starch dust explosion accidents more effectively, Liang et al. studied its oxidation characteristics. Six kinds of gases were detected by EGA-FTIR and provided a theoretical basis for preventing and controlling explosion accidents [288]. An innovative screening platform based on several thermoanalytical and spectroscopic methodologies was developed and validated for the characterization of different milks [289], wines [290] and for the on-site detection of cannabinoids in hemp seed oil [291,292,293] for food safety control of commercial products.

Direct pyrolysis of waste bread under a CO_2_ atmosphere allowed one to prepare an activated carbon foam. The preparation process was investigated online by EGA-FTIR that provided a reference for the utilization of waste bread as a promising precursor of activated carbon foam adsorbent, which has a highly porous structure and excellent floatability in water [294].

The influence of the atmosphere on the decomposition of vegetable oils was proposed by means of the profiles and evolution of gaseous products by de Carvalho and coworkers [295].

## 25. Conclusions

The on-line thermally induced evolved gas analysis by Fourier-transform infrared spectroscopy (OLTI-EGA-FTIR) is a useful tool to determine the quantitative parameters of each thermally induced reaction in the sample under examination and to confirm the nature of the products released as a function of the temperature. This information makes it possible to objectively test the proposed mechanisms.

The main limitation of the EGA-FTIR technique is the minimum amount of sample needed to obtain a good spectral signal. Often, as evidenced by several cited references, the coupling involves multiple simultaneous instruments, such as, for example, EGA-FTIR-MS or EGA-FTIR-GC-MS. These analytical solutions significantly increase the detection efficiency.

The growing number of publications related to EGA-FTIR and EGA-FTIR-MS coupling demonstrates the flexibility of this analytical approach, which allows for its application to different matrices.

## Data Availability

Not applicable.

## Abbreviations

EGA	Evolved gas Analysis
TG	ThermoGravimetry
TGA	ThermoGravimetric Analysis
MS	Mass Spectrometry
QMS	Quadrupole Mass Spectrometry
FTIR	Fourier-Transform InfraRed spectroscopy
UV	UltraViolet spectroscopy
XRD	X-Ray Diffraction
PMMA	Polymethylmethacrilate
DART	Direct analysis in real time
DFT	Density Functional Theory
DSC	Differential Scanning Calorimetry
DTA	Differential Thermal Analysis
ICP-OES	Inductively Coupled Plasma—Optical Emission Spectroscopy
MOF	metal Organic Framework
DRIFTs	Diffuse Reflectance Infrared Fourier-Transform Spectroscopy
SEM	Scanning Electron Microscopy
HRTEM	High-Resolution Transmission Electron Microscopy
FID	Flame Ionization Detector
HS-GC-MS	Head Space GasChromatography/Mass Spectrometry
Py-GC/MS	Pyrolysis-GasChromatography/Mass Spectrometry
PCA	Principal Component Analysis
OLTI-EGA	On-Line Thermally Induced Evolved Gas Analysis
NMR	Nuclear Magnetic Resonance
